# Medical and Philosophical Intelligence

**Published:** 1818-02

**Authors:** 


### MEDICAL AND PHILOSOPHICAL INTELLIGENCE.

[The following paper arrived too late for insertion among the Original Communications in the beginning of the Number.]

*To the Editors of the London Medical and Physical Journal.*

GENTLEMEN,

IF you think the following few remarks worth inserting in your valuable Journal, they are much at your service.

Some months ago, travelling in the north of England, I stopped a few days near Doncaster, where I saw a poor man apparently suffering under the most excruciating pain. Upon enquiring into the circumstances of the case, I was informed, that he had lain down to sleep upon some grass, after working hard the whole morning, and that he had suddenly awoke, screaming out that something had bitten him. On looking at the left arm, there was a small black mark; his countenance had a dejected and cadaverous appearance; and, his pulse between forty and fifty, small and fluttering. I had no doubt that an adder had bitten him; and, from having had many natives of India brought to me labouring under nearly the same symptoms, from the bites of different kinds of snakes, and which were invariably relieved by the use of the liquor ammoniæ, when it had been used in time, I immediately enquired if any of the neighbours had any spirits of hartshorn, which, fortunately, was procured in a few minutes; I also got some gin (although brandy is the spirit I have generally been in the habit of giving,) and, with difficulty, could get the man to swallow about half an ounce of the spirit mixed with a little water, and about a drachm of the alkali. The effect was as sudden as I have ever seen it. In about ten or fifteen minutes, the patient's eyes became more bright, his pulse fuller and stronger, and his countenance altogether more cheerful; and, by the repetition of the same dose as above stated, in about the space of an hour and a half, he appeared perfectly recovered. I left another dose to be taken at ten o'clock at night; and, in the morning, when I again saw him, he said he was quite well, but a little numbness and weakness of the arm, for which he was ordered to have it rubbed with linamentum volatile. The third day after, he returned to his work, and was quite well. I am, gentlemen,

Your most obedient servant,

15, *Grove, Bath;* *Jan.* 12, 1818.

P. LESLIE, M.D. Mem. Coll. Surg. Lond. E. I. Comp. Med. Estab. Bombay.

---

We have received several practical communications from Mr. Walker, of Oxford. Many of them are certainly not new in London, nor to gentlemen who have recently finished their studies in London; yet none are without their use. We shall endeavour to offer as many of them, and in as few words, as our limits and perspicuity will admit.

On the subject of Linseed Cataplasms, Mr. W. has several useful remarks:—that, though generally adopted and convenient, on account of the facility with which they are spread, and retain their form, yet, for irritable sores, they are too harsh in their substance, and too unyielding in their figure. We have frequently found this the case, when applied to the nipples or glans penis. We would also add, that this is not always sufficiently attended to in hospitals; though, in private practice, our patients insist on being heard, and here we are often obliged to refer to the bread and milk poultice. Where the skin is entire, and not highly inflamed, Mr. W. prefers the linseed poultice. Mr. W. insists on this the more from having found some practitioners attend only to internal remedies, when the ill condition of the sore has been induced and kept up by the harshness of the linseed poultice. With a change of dressing, he has often found it useful to discontinue the internal remedies; the reiteration of which has prevented the patient from taking wholesome nutriment. He is equally an enemy to over-stimulating diet, or wine given merely as an antiseptic.

In addition to his remarks in our Journal for June 1815, Mr. W. adds, " I forbore at that time giving a direct opinion respecting the best composition for adhesive plaster: I can now offer, with confidence, what I conceive to be complete in that respect.—Thus, for ordinary application, I use the following:—of the emplastrum plumbi, of the present London Pharmacopœia, ℔i, and of the Burgundy pitch, ℥ss; increasing the proportion of the Burgundy pitch, when required to be more stimulating or more adhesive. The emplast. plumbi should be of a sufficiently hard consistence, without being brittle, to form, with the Burgundy pitch, a plaster of sufficient tenacity, without being too hard or harsh to the sore; and, indeed, its degree of hardness should be varied or adjusted according to the nature of the sore.

" For want of these circumstances being attended to, I have occasionally seen ordinary sores, especially in inflammatory and irritable habits, brought into a very painful and ill-conditioned state, entirely from the dry harsh nature of the plaster; which is sometimes the case in the adhesive plaster, procured ready spread by the wholesale manufacturers of the article.

" The plaster may be, and often is, spread too thin. The Irish cloth, calico, or black silk, (each of which have their peculiar advantages according to circumstances,) should be covered with an uniform stratum of plaster, of a slight but determined thickness. In the use of adhesive plaster to sores, which, when judiciously made and applied, is incomparably the most effective application, it is occasionally necessary, if undue heat occur, to cover the plaster with cloth of one or more folds, and renewed occasionally, moistened with cold water. Cold, by reducing heat, produces good effects in local inflammation of every kind; and, when used with judgment and due caution, in particular cases, its benefit is incalculable: yet it is often neglected where it would be highly grateful and salutary, through ignorance or timidity. In gout and erysipelas, I have applied cold so far as to moderate the violence of the attack, but never ventured so far as to attempt to subdue, or suppress it solely, by that means.—RICHARD WALKER."

---

Since our account of Miss M'Avoy, by Dr. Renwick and Mr. Sandars, was concluded, we have received a long communication from a most respectable quarter in Liverpool. Of this we have only room for the concluding part:—

"Mr. Sandars' pamphlet has certainly produced, as must be supposed, a considerable effect upon those who have not seen Miss M'A. and upon many who have seen her but once or twice; and particularly upon those who have gone to see her, but have been disappointed by her not having the power, which has been very frequent. But, surely, the spirit in which it was evidently written is too unphilosophical to make much impression upon philosophical people. Full, fair, and persevering attention and experiments ought alone to satisfy reflecting persons. Dr. Renwick continues his observations, and probably will give the world a continuation of the Experiments. Miss M'Avoy has now been very ill many weeks: she has had dreadful convulsions, the similar discharge of fluid from the brain, &c. She has now occasional convulsions every day, a most violent pain in the region of the heart, and has not the use of her faculty in the smallest degree. A gentleman has proposed to her to go to London, and to bear her expences: she has accepted the offer with great willingness. That she has received money is erroneous altogether. Some papers have appeared in some of the last numbers of the Liverpool Courier, which I advise you to peruse; as well as a paper in the Cheltenham Gazette, of the 31st December. You must excuse the very confused manner of my writing, as I am preparing to set off early tomorrow morning."

We are glad to find Dr. Renwick is continuing his experiments. Though we could wish Mr. Sandars had written in milder temper, we are better pleased with his open statement of objections, than with those mealy-mouthed characters who, though on the spot, content themselves with condemning the whole as an imposture, without examination, or without assigning any reason. When objections are stated, experiments may be varied accordingly; and, most of all, we advise the utmost tenderness and urbanity in every part of the controversy. By such treatment, Miss M'Avoy and her friends, should they prove to be in error, will be the more ready to acknowledge it, and truth be more easily elicited. We do not recollect any want of propriety in Dr. Renwick's language.

Since the above was composed for the press (22d Jan.), we have received a communication, which imposes a most painful task upon us. The peculiarity of Miss M'Avoy's faculty might fairly authorise a doubt of its existence, and even an assertion of its impossibility, according to any of the known laws of optics or living animal matter. But, to do this with severe personal expressions on the authority of one party in contradiction to another, was at least unnecessary, to use no stronger term, especially when the means of information were at hand; or, if they were not, we are not made acquainted with the difficulties.

As it is evidently the wish of our correspondent that his letter should appear this month, and as there is not time for an answer to any request we may send to Liverpool, we have taken the liberty of inserting only an extract, with no other alterations than a few words, necessary in consequence of the omission of other passages.

"I wish the editor of —— had called on me, previous to the publication of this severe judgment concerning Miss M'Avoy. It would have given me much pleasure to have introduced him, and he might then have had an opportunity of judging better than by taking the ex-parte evidence of gentlemen who have seen very little of Miss M'Avoy. In the narrative, there is an account of the examination which took place at Wavertree, taken from the memoranda of Dr. Jardine, when Dr. Trail and Dr. Vose were present; and of another visit which Dr. Trail paid in company with Mr. Brandreth, Mr. Bickerstaff, and myself, to which I refer those persons who wish to be informed on the subject. Dr. Vose visited her once, several months ago, when, I believe, from being in a state of insensibility, she was unconscious of his visit. From these examinations, they have formed a judgment of Miss M'Avoy, which, I am convinced, is erroneous. A great deal has been said, in different periodical publications, tending to prejudice the public mind against her, which are partly derived from the extraordinary nature of the case, but chiefly from extracts taken from Mr. Sandars's pamphlet, which have been disseminated with great assiduity by the partizans enlisted against Miss M'Avoy. Had these observations been merely confined to the newspapers of the day, I should have declined taking any notice of them, as my time will not allow of my entering into unnecessary discussion on this subject; but, as they are now embodied in a respectable periodical publication, it becomes my bounden duty to protest against opinions thus foisted upon the public. As a gentleman, I pledge myself to the truth of the facts stated in the narrative. In the composition of that narrative, I allow there are several typographical and other errors, which may be excused, upon the principle that it was issued from the press very hastily to meet the wishes of the public. Mr. Sandars has noticed them with a spirit of which I do not envy him the possession, and he is welcome to all the advantage he can make of them. Whether he has succeeded, or not, in his object, the public will judge; but, what will be the consequence of the assertions of himself, and of the other gentlemen, when it is proved that Miss M'Avoy is really blind?—I have reason to believe, this will be the case as soon as she is examined by men, unbiassed by the prejudice which prevails chiefly amongst people who have never seen her.

"Miss M'Avoy has been very indifferent in health for nearly three months. On the 15th of November, she was seized with convulsions, which terminated on the evening of the 26th, after the discharge of a quantity of fluid,—a small part of which was forwarded to Sir J. Banks, and another part to Dr. Monro, of Edinburgh. On the 29th, the convulsions came on in a slighter degree in the evening, and returned, more or less, during each night, until the 29th of December, when they disappeared; but a considerable difficulty of breathing, which came on in the night, as the convulsions ceased for a time, with a similar affection about the region of the heart, to that mentioned in the narrative, still continued. In the last fifteen days, she has been bled four times, with considerable relief each time. Yesterday, she was much better; but, to-day, she is worse again. The right side is perfectly paralytic, and the peculiar feeling in the right foot, upon any sudden pressure, which came on during the convulsions, still continues. She is obliged to be supported in getting into bed. In the day she sits up in her chair, and employs herself in sewing, or in making paper-ornaments. Her food is chiefly coffee, milk, and sugar. She says, she obtains little or no sleep, yet she does not look thin in the face. Her mother observes, that she appears to waste in the throat of the right side, and in the right breast. There is an appearance of an enormous swelling, particularly in the lower extremities, which is more considerable in the evening.*

"If you think it proper to publish this letter in the Journal for February, it will be a sufficient contradiction of the reports in circulation, of Miss M'Avoy's case being a *gross imposition upon public credulity.* I have the honour to be, &c.

"THOMAS RENWICK."

As we have professed to devote this Number very much to optical purposes, it cannot be thought intrusive if we offer a few words. We confess it gives us some pain to find that a town, which can boast an Allison, a Currie, a Roscoe, and (we believe) a Clarkson, at variance whether a young lady is blind or not. When we reflect on those stupendous monuments of enterprise, their docks, in which they anticipated Hull and London; their Exchange and its appurtenances, in which they greatly excel us; the Athenæum and Lycæum, in which they have led the way to all we gain from our Institutions; not to mention their Asylum for the Blind, in which also they were before-hand with the metropolis:—when we reflect on all these, we cannot help being surprised at the manner in which this controversy has been carried on. By one party, with a rudeness which nothing will excuse; by the other, with a sensitiveness which the very conduct of their adver-

"* The power of distinguishing colours scarcely occurs at present, but she has once or twice named a colour within the last four days."

saries might have blunted. Distant as we are from the scene, and highly as we respect all those on either side with whom we have any acquaintance, we cannot be expected to do more than offer a few hints, which we trust will not be ill received.

As Miss M'Avoy does not pretend always to possess this faculty, it may fairly be urged, that her failure at particular trials is not sufficient evidence against others in which she has succeeded better. But, among so many philosophers, how is it that we have so few conjectures concerning the nature of her complaint; and, when that is investigated, will it lead to any elucidation of her real condition?

From the discharge by the nose, it is not unreasonable to suspect hydatids, which, when entire, may so press on the optic nerve as to induce blindness. It is now pretty generally admitted, that these substances are animals, consisting only of a bag and its fluid contents; that they possess a life independent of the subject in which they grow, excepting that, like parasyte plants, they draw their nourishment from the surrounding living matter. Under this state of blindnes, Miss M'Avoy has learned, like many others, to amuse herself with feminine employments. By long habit, her fingers became the organ by which only she had any knowledge of her operations. Let us suppose, after a very long continued habit of this kind, that one or more hydatid burst, and that the fluid issuing from it escaped in the manner described. If, by the cessation of the former pressure, she now recovered an imperfect vision, might she not, on this sudden but imperfect recovery, impute all the advantages she derived from it to those organs by which only she had for some time been accustomed to judge of the figure and solidity of objects; and, if this power of ascertaining their colour and shades also should prove only transitory, might not the difficulty of associating these appearances with the senses peculiar to each be still greater? We well know the varying form of hydatids, their uncertain growth, and death; and may not this variety and uncertainty have enabled the patient at one time to see imperfectly; at another, have disabled her altogether. May not the same influence have extended to the eustachian tube; and the bursting or death of the hydatid not only have left that passage clear, but, by exciting slight inflammation, have rendered the whole organ more sensitive than before, and thus account for her hearing being so peculiarly acute after the discharge? The offensive state of the fluid is the constant condition when an hydatid dies; the flakes were probably the tunics.

It seems unnecessary to say any thing concerning the convulsive and paralytic affections, or pains in various parts of the head. The growth or bursting of hydatids, and the consequent partial pressure or diffusion of fluid on different parts of the brain, is sufficient to account for all these symptoms. The absorption of the fluid may explain the convalescence, and the increase of other hydatids the subsequent return of the symptoms.

The great difficulty, we admit, is, that a person who has seen should be ignorant of the organ by which the impression of colours is received. It is true, if both organs were permanently to remain perfect, the association must be gradually restored. But we are not always aware how easily we may sometimes be deceived. Why are we so anxious to watch the lips of those whom we hear only imperfectly? Because, by the motion of the lips, we can often complete a sentence which we only hear imperfectly. Yet few of us could ascertain how much we learn by our ears, and how much by our eyes.

Still it will be urged, that our conjecture, if just, will be insufficient to account for the full faculty that Miss M'Avoy is supposed to possess. We grant it; but we offer it as a consideration to both parties, and most of all to those who suspect her of a crime we would scarcely believe at any age, but particularly in so young a subject,—namely, that of deceiving her confessor. Whatever may be thought of that officer and his duties, we have seen many instances of advantages from them, and are unwilling to believe it could be abused on this occasion.

Having offered these hints, we wait with much patience for further information; at the same time trusting, that, should the disease prove fatal, or any other event shorten life, a satisfactory examination will follow.

---

The following papers should have been added to those we collected in a former Number, on the health of the metropolis.

*To the Editor of the Times.*—Sir,—Convinced that I discharged a public duty by attempting to allay an unnecessary alarm, I published a letter a few days since, in which I briefly said, London is scarcely ever free from typhus-fever; its extent or malignity has not been increased at any period during the present year; and, for the truth of these assertions, I confidently appeal to the candour of my brethren in the profession.

I have twice been contradicted without much ceremony. The first of my assailants neither identified his profession, his christian name, or his residence, and, therefore, has but little cause to complain, if his remarks remain unnoticed. But, having appealed to my brethren in the profession, I am bound to notice a letter of Dr. Bateman's, which appeared in Friday's paper, commencing with a mis-statement of my words, and an observation which calls in question my opportunities of being acquainted with the subject in dispute. The doctor may rest assured that, although, according to his letter, I reside in the west-end of the town, my door is ever open to the poorest class of patients, and my solicitudes in their behalf have extended to St. Patrick's Female School, where three hundred children are educated, in her own neighbourhood, by a benevolent lady who has permission to send even the parents to my house for advice. If necessary, I hope I could dilate upon this part of the letter; but I shall briefly remark, that the writer might have joined issue with me more fairly, than by attempting to prove the existence of a malignant fever, by the extension of a very mild epidemic, which is acknowledged to be only a synochus, or simple continued fever. With every disposition to be grave, it is difficult to forget the anecdote of the tyro, who asserted that his patient must have devoured a horse, because he saw a saddle lying under the bed.

Unfortunately for the doctor, the very article which followed his long letter, contained an official declaration from Mr. Wakley, of Guy's Hospital, that, on the 21st instant, he visited four of the principal hospitals, and found they had not ten cases of typhus-fever.

I have visited the Fever Institution, as well as some gentlemen who are acquainted with its management, and ascertained the following facts. So many cases of mild fever have been intruded upon the charity, by the facility of acquiring erroneous certificates, that Dr. Bateman had determined on representing the evil to the committee, and calling for regulations which, in future, might prevent it. The extension and increased publicity of the building has increased the number of patients, and the parish work-houses have transferred a share of their unprecedented burdens to it. None of the visitors have carried away infection; and the surgeon-apothecary had prepared an honest statement for the public, which, owing to some unavoidable circumstance, has been delayed. This, I hope, may yet appear; and the advisers of the crown may adopt my humble recommendation to establish a permanent board of health, for the prevention of unnecessary alarms, as well as of the ravages of malignant fever, for the future.

*Cavendish-square;* I am, yours, &c.

*Oct.* 26. JOHN BRINE, M.D.

*To the Editor of the Times.*—Sir,—Having good reason to believe the mode of treating this fever by the cold affusion is, unfortunately, too little pursued by the generality of medical practitioners, perhaps through fear, or for want of still further proof of the success of such treatment; I beg, through the medium of your paper, to lay before the public the following few particulars, which may possibly tend to encourage practitioners, even in the most hopeless cases, to resort to this plan. In the neighbourhood of Aldgate, this fever has lately shown itself in different families with some considerable degree of violence. In three cases out of several which have come under my care, the cold affusion was had recourse to with success; and, the following case, I think, merits some attention, as it goes to prove, that at any period of this fever it may be employed with advantage. On Thursday, the 16th inst. at eleven o'clock at night, I was called to attend a female patient in Jewry-street, whom I found in a state of complete insensibility, apparently breathing her last, and without the most distant prospect of her recovery: the pulse so low as not to be felt; the tongue, mouth, and teeth, covered with a black thick fur or crust, and completely parched; the breath highly fœtid; the heat of the body below the natural temperature; the lower extremities cold; in short, she appeared on the very point of dissolution. In this hopeless state, I suggested the idea of the cold affusion being tried, even at this very advanced period (being the twelfth day of the disorder) to which her friends readily agreed, and which was accordingly used with the happiest effect; the pulse immediately rose, the senses returned, a general warmth and slight determination to the skin ensued, the tongue became moist, and from that hour she continued to recover, and is now able to go out.

22, *Jewry-street*, *Aldgate*; I am, Sir, your obedient servant,

*Oct*. 27. E. B. MORGAN.

[We understand Mr. Morgan's method of using the cold affusion was as follows:—The patient being placed in a chair, he took a vessel, containing about two gallons of cold water, which he poured suddenly over the whole body. The patient was wiped dry, and immediately put into bed, and a little warm wine and water administered.]

---

Dr. Francis Moore, of New-England, proposes, in cases of ophthalmia which resist the ordinary mode of treatment, that the disease should be treated like others known to arise from hepatic diseases. On some chronic cases, supposed to arise from weakness in the vessels of the conjunctiva, and for which astringents are usually administered, he remarks,—

"However plausible the operation of astringents may appear in theory, they frequently disappoint the expectation of the practitioner. In reflecting on this subject, I think more has been expected of them than is in their power to perform. To produce any important effect, astringents must be long applied to the part, but who does not know, that any extraneous matter introduced into the eye, is immediately washed out by an increased flow of tears. Dr. Barton, speaking of chronic ophthalmy, observed, 'I know of no remedy on which much reliance can be placed.'

"From the failure of astringents, I was led to try the means used for curing varicose veins in other situations, and, for this purpose, had recourse to the expedient of tying a folded silk handkerchief, moderately tight, across the eyes at bedtime, to be worn during the night. By this means, all light is excluded, the eye is gently fixed in the orbit, and the slight degree of compression gives the vessels an opportunity of recovering their tone. The experiment has answered my expectation, and, from the number of cases in which I have used it successfully, I can recommend its adoption.

"The importance of this remedy will be better understood, when we consider that, in this enlarged state of the vessels of the conjunctiva, the eye-lids are imperfectly closed; so that sufficient light is admitted to be a cause of irritation. In sleep (which is the repose of the senses) the eye, in a diseased state, cannot fully participate; it is exposed to the stimulus of light *ab extra*, and is obedient to fancy in dreams, by which, during the night, a considerable degree of rotation of the eye subsists, in this imperfect sleep; and the subject rises in the morning with all his symptoms increased. During our waking hours, the elevation of the eye-lid relieves the eye from the full degree of irritation in its rotatory motion, which it experiences in its movement in the night when closed. By the means above recommended, all light is excluded, and the eye is preserved in a state of perfect repose, and, of course, is relieved from all irritation."

Mr. Leigh, in his "Journey in Egypt," gives the following account of the treatment of this disease among the natives:—"On the first approach of inflammation, an Arab binds a handkerchief round his eyes as tightly as possible, and excludes light and air during three days and three nights; after which, he removes the bandage, and resorts to frequent bathing with cold water. This method is generally successful; but it is worth noting also, that relief will be obtained by introducing a small quantity of powdered sugar between the eye-lids at night."—Probably the last remedy is for the chronic state of the disease.

---

*Mr. Taunton's Report from the City of London Truss Society.*—The following statement of the situation and occurrence of Hernia, at different periods of life, has been extracted from the register of the patients relieved by the City of London Truss Society, within ten years.

In 15,001 cases, 12,231 were males, and 2,770 were females.

| Males. | Females. | | | | |
|---|---|---|---|---|---|
| 2,344 | 52 | Left inguinal | 6677 inguinal | 7702 single | |
| 4,230 | 51 | Right inguinal | | | |
| 70 | 419 | Left femoral | 1025 femoral | | |
| 78 | 458 | Right femoral | | | |
| 5,016 | 44 | Double inguinal | | 5430 double | |
| 65 | 305 | Double femoral | | | |
| 74 | 590 | Umbilical | | | 787 |
| 36 | 87 | Ventral | | | |
| 1 | 2 | Perineal | | | 3 |
| 1 | 3 | Obturator | | | 4 |
| 19 | 32 | have undergone operations, all except *one* have been completely successful | | | 51 |
| 190 | 152 | with umbilical and inguinal hernia have been cured | | | 342 |
| 101 | 27 | with prolapsus ani | | | 128 |
| | 546 | with prolapsus uteri | | | 546 |
| 6 | 2 | with varix of the abdominal veins | | | 8 |
| 12,231 | 2770 | 15,001 | | | 15,001 |

| | | | | | |
|---|---|---|---|---|---|
| 1,127 | Patients were relieved with trusses under 10 years of age. | | | | |
| 748 | ditto, | between | 10 and | 20 | ditto. |
| 1,515 | ditto, | ——— | 20 and | 30 | ditto. |
| 2,460 | ditto, | ——— | 30 and | 40 | ditto. |
| 2,685 | ditto, | ——— | 40 and | 50 | ditto. |
| 2,562 | ditto, | ——— | 50 and | 60 | ditto. |
| 1,795 | ditto, | ——— | 60 and | 70 | ditto. |
| 590 | ditto, | ——— | 70 and | 80 | ditto. |
| 79 | ditto, | ——— | 80 and | 90 | ditto. |
| 4 | ditto, | ——— | 90 and | 100 | ditto. |
| 13,565 | | | | | |

Eight females each had umbilical and double femoral hernia, 1 female had double ventral on the right side and left femoral hernia, 2 females had large ventral and double femoral hernia, 6 females had umbilical and ventral hernia, 1 female had right femoral hernia and prolapsus uteri, 1 female had prolapsus uteri and prolapsus ani, 1 female had ventral hernia and prolapsus uteri, 1 female had umbilical hernia and prolapsus uteri, 8 females had umbilical and single femoral hernia, 5 females had right inguinal congenital hernia, 7 females had left inguinal and right femoral hernia, 1 female had left femoral and right obturator hernia, 5 females had ventral and single femoral hernia, 1 female had double femoral and right inguinal hernia, 6 females had double inguinal and umbilical hernia, 2 females had right inguinal and umbilical hernia, 1 female had right inguinal and ventral hernia, 7 females had left inguinal and left femoral hernia, 1 female had left femoral hernia outside of the femoral vessels, 1 female had right femoral hernia outside of the femoral vessels.

Thirty-one males each had left femoral and right inguinal hernia, 9 males had left inguinal and left femoral hernia, 23 males had left inguinal and right femoral hernia, 11 males had double inguinal and right femoral hernia, 9 males had double inguinal and left femoral hernia, 6 males had right inguinal and right femoral hernia, 2 males had double inguinal and double femoral hernia, 6 males had double femoral and right inguinal hernia, 3 males had ventral and right inguinal hernia, 1 male had ventral and right femoral hernia, 2 males had ventral and left inguinal hernia, 3 males had ventral and double inguinal hernia, 1 male had double ventral and double inguinal hernia, 1 male had left inguinal, umbilical, and ventral hernia, 1 male had double inguinal, umbilical, and ventral hernia, 1 male had umbilical and left inguinal hernia, 3 males had umbilical and right inguinal hernia, 1 male had umbilical and left femoral hernia, 1 male had umbilical and right femoral hernia, 11 males had umbilical and double inguinal hernia, 2 males had double femoral and right inguinal hernia, 1 male had right femoral hernia outside of the femoral vessels, 1 male had double inguinal hernia and prolapsus ani.

Seven hundred and fifty-four patients had congenital hernia.—The last were principally infants, who were permanently relieved. In all the adults, the testicle was found diminutive, and formed as if when it descends no lower than the groin.

---

*London Medical Society, Bolt-court.*—This Society resumed their meetings on the last Monday in January; when the paper on Muscæ Volitantes, contained in our original department, was read. A paper by Dr. Clutterbuck, on Fever, followed;—both these communications were particularly interesting, from the cases having occurred to gentlemen of the faculty. Mr. Blegborough was the subject of the fever, attended by Dr. Blegborough and Dr. Clutterbuck. All the parties being present, the conversation which followed could not but prove particularly instructing.

### BILLS OF MORTALITY.

### *Diseases and Casualties for the Year* 1817.

| DISEASES. | |
|---|---|
| Abortive & Stillborn | 700 |
| Abscess | 98 |
| Aged | 1675 |
| Ague | 2 |
| Apoplexy & Suddenly | 462 |
| Asthma | 743 |
| Bed-ridden | 5 |
| Bile | 6 |
| Bleeding | 45 |
| Bursten & Rupture | 43 |
| Cancer | 99 |
| Chicken-pox | 1 |
| Childbed | 252 |
| Colds | 14 |
| Colic, Gripes, &c. | 7 |
| Consumption | 4200 |
| Convulsions | 3242 |
| Cough and Hooping Cough | 645 |
| Cow-pox | 1 |
| Cramp | 1 |
| Croup | 109 |
| Diabetes | 3 |
| Dropsy | 718 |
| Epilepsy | 5 |
| Evil | 6 |
| Fevers of all kinds | 1299 |
| Fistula | 3 |
| Flux | 9 |
| French Pox | 86 |
| Gout | 54 |
| Gravel, Stone, Strangury | 24 |
| Grief | 4 |
| Headmoldshot, Horseshoehead, & Water in the Head | 419 |
| Imposthume | 2 |
| Inflammation | 1002 |
| Jaundice | 75 |
| Jaw Locked | 2 |
| Livergrown | 76 |
| Lumbago | 1 |
| Lunatic | 244 |
| Measles | 725 |
| Miscarriage | 6 |
| Mortification | 304 |
| Palpitation of the Heart | 4 |
| Palsy | 162 |
| Pleurisy | 22 |
| Purples | 1 |
| Quinsy | 2 |
| Rash | 2 |
| Rheumatism | 11 |
| Scrofula | 5 |
| Scurvy | 6 |
| Small-pox | 1051 |
| Sore Throat | 5 |
| Sores and Ulcers | 11 |
| Spasm | 25 |
| St. Anthony's Fire | 7 |
| St. Vitus's Dance | 1 |
| Stoppage in the Stomach | 24 |
| Stricture | 1 |
| Swelling | 1 |
| Teeth | 449 |
| Thrush | 111 |
| Tumor | 3 |
| Water in the Chest | 68 |
| Worms | 12 |

| CASUALTIES. | |
|---|---|
| Broken Limbs | 6 |
| Bruised | 2 |
| Burnt | 41 |
| Choaked | 2 |
| Drowned | 119 |
| Excessive Drinking | 12 |
| Executed | 10 |
| Found Dead | 29 |
| Fractured | 4 |
| Frighted | 9 |
| Killed by Falls & several other Accidents | 65 |
| Killed themselves | 34 |
| Murdered | 3 |
| Poisoned | 6 |
| Scalded | 4 |
| Shot | 1 |
| Starved | 8 |
| Strangled | 1 |
| Suffocated | 11 |

| | | | |
|---|---|---|---|
| Christened | Males | 12624 | In all 24129 |
| | Females | 11505 | |
| Buried | Males | 10033 | In all 19968 |
| | Females | 9935 | |

Whereof have died,

| | | | |
|---|---|---|---|
| Under Two Years of Age | 5698 | Sixty and Seventy | 1614 |
| Between Two and Five | 2019 | Seventy and Eighty | 1224 |
| Five and Ten | 929 | Eighty and Ninety | 683 |
| Ten and Twenty | 706 | Ninety and a Hundred | 156 |
| Twenty and Thirty | 1364 | A Hundred | 7 |
| Thirty and Forty | 1795 | A Hundred and One | 0 |
| Forty and Fifty | 1983 | A Hundred and Three | 0 |
| Fifty and Sixty | 1788 | A Hundred and Five | 2 |

Decreased in the Burials this Year 348.

There have been Executed in the City of London and County of Surrey 26; of which Number 10 only have been reported to be buried within the Bills of Mortality.

In the Report of Diseases for the last month, we generally remarked, that the number of fevers is exactly similar to the preceding year, which would lead us to suppose that, however common typhus may have been, it has not been often fatal; neither has the number considerably increased towards the close of the year.—The deaths by small-pox progressively increase. In the year ending December 10th, 1816, they were 653; in the last year,

1051. This is what has often been suggested would be the case as soon as the laudable zeal for vaccination ceased in the country: and, in consequence, young people would arrive in the metropolis susceptible of the variolous poison. That such is the cause is the more probable, as the number vaccinated at the Small-Pox Hospital exceed by a thousand those of the former year. It is strange that the public has yet to learn that the danger of the small-pox spreading is not according to the diffusion of the infection in London, where it is every-where diffused, but according to the number of subjects susceptible of the disease. The deaths by measles are diminished from 1106 to 725; but even the latter is great compared to what occurred before the number by small-pox was so much lessened. Of the whole number, nearly a fourth have died under two years, and a tenth between two and five.

*Hospital for the Small-Pox, for Inoculation and for Vaccination, at Pancras.*

An account of the numbers of Patients admitted; also the Out-Patients, and the Mothers admitted to nurse their Children; from January 1, 1817, to January 1, 1818.

| 1817. | With the casual Small-Pox. | For Inoculation. | For Vaccination. | Out-Patients for Vaccination. | Mothers. |
|---|---|---|---|---|---|
| January | 18 | 0 | 0 | 81 | 0 |
| February | 11 | 2 | 0 | 168 | 1 |
| March | 7 | 7 | 0 | 316 | 1 |
| April | 11 | 2 | 0 | 419 | 1 |
| May | 15 | 8 | 0 | 366 | 1 |
| June | 27 | 6 | 0 | 445 | 0 |
| July | 17 | 5 | 0 | 362 | 3 |
| August | 7 | 1 | 0 | 268 | 0 |
| September | 12 | 1 | 0 | 334 | 0 |
| October | 15 | 4 | 2 | 179 | 2 |
| November | 14 | 1 | 1 | 121 | 0 |
| December | 6 | 5 | 0 | 65 | 0 |
| | 160 | 42 | 3 | 3124 | 9 |
| Died | 48 | 1 | none. | none. | none. |

Increased number with casual small-pox ........................ 19
—— ditto for inoculation ........................ 5
—— ditto out-patients for vaccination ............. 811
Decreased ditto in-patients for vaccination............... 2

By order of the committee,

John Christian Wachsel,

*Jan.* 1, 1818. Resident Apothecary, &c. &c.

After the favourable account of vaccination before given from the above Hospital, we were greatly surprised to find in Mr. Field's Reports of Christ's Hospital, the accuracy of which cannot be questioned—

"The small-pox has been unusually frequent with us. Four cases have occurred in boys who have never been inoculated, and

*seven* after vaccination: the former have been somewhat confluent, but all have recovered. The latter were, as usual, very mild, with the exception of one case, in which the eruption was very copious, and its termination protracted, but without any reason to apprehend danger."

What renders this report the more remarkable is, that the annals of that valuable Institution do not, we believe, furnish more than one case of casual small-pox after variolous inoculation.

---

THE following account of the effects of thunder is an interesting fact in Physiology. The event happened six miles from Dublin. Two children were severely stunned, and one remains deaf, the hair in some parts singed, and several marks on the skin, as if made with a hot iron; the other child recovered. In an adjoining cabin, three cows were found dead. When the spot was visited two days after their hides had been taken off and the bodies dismembered, the latter emitted an extremely offensive odour, and the blood was said to be in a fluid state. Hence we may conclude, that absolute universal death instantly took place,—that the blood never coagulated,—and probably the muscles never stiffened, as putrefaction came on so early in the winter season.

---

As an article of Intelligence, we have received an anonymous report, that "a boy, seven years of age, *born* deaf and dumb, has been *restored to the use* of hearing and speech." This remarkable *restoration* of a faculty never before possessed, we should not have noticed, had not the same appeared in the public prints, and in a manner as if attested by several of the most respectable physicians and surgeons in the metropolis. In our opinion, it becomes them to give the particulars of this most extraordinary case, with their own signature, or to explain the manner in which their name has been used.

---

A remarkable case of Hydatids between the tables of the skull has lately occurred at St. George. The tumour was successfully taken away by Mr. KEATE.

---

Dr. GRANVILLE, the successful candidate for the office of Physician-Accoucheur to the Westminster Dispensary, gained his election by a small majority. The applications on both sides were immense; and the venerable President of the Royal Society was induced to attend on this occasion. The resignation of Dr. Thynne, though in favour of the other candidate, it is supposed, was serviceable to Dr. G.—We know not to what we are to impute it, that no one of the candidates made the usual addition to such applications, of the name of the physician he wished to succeed; and both of them took much pains to assure the public that they had no claims on the score of officiating for him. The gentleman was, we believe, the author of a new practice in "the treatment of uterine hæmorrhage."

---

Professor BRAND is delivering a course of Lectures on Poisons, at Apothecaries' Hall. We have encouragement to hope they will be published.

### A METEOROLOGICAL JOURNAL,

By Messrs. William Harris and Co. 50, Holborn, London.

*From the 20th November to the 19th December, inclusive.*

| Day of Month. | Moon. | Rain gauge | Therm. | | | Barom. | | De Luc's Hygrom. Dry | | Damp | | Wind. | | Atmospheric Variation. | | |
|---|---|---|---|---|---|---|---|---|---|---|---|---|---|---|---|---|
| Dec. | | | | | | | | | | | | | | | | |
| 20 | | | 41 | 43 | 31 | 29·45 | 29·61 | | | 15 | 15 | N | E | Fine | | |
| 21 | | | 34 | 37 | 32 | 29·53 | 29·55 | | | 5 | 5 | NE | ENE | Clo. | Fine | Clo. |
| 22 | | | 32 | 31 | 32 | 29·62 | 29·63 | | | 5 | 5 | N | N | Fine | | Clo. |
| 23 | ○ | | 32 | 32 | 27 | 29·65 | 29·69 | | | 5 | 15 | N | N | Fine | | Snow |
| 24 | | | 28 | 31 | 27 | 29·74 | 29·81 | | | 10 | 9 | N | N | Fine | | |
| 25 | | | 30 | 32 | 28 | 29·94 | 30·04 | | | 9 | 10 | N | NNW | Fine | | |
| 26 | | | 30 | 31 | 32 | 30·00 | 30·30 | | | 15 | 10 | N | SSW | Fog | Fine | |
| 27 | | | 35 | 37 | 32 | 29·60 | 29·54 | | | 10 | 8 | SW | SSW | Clo. | | Rain |
| 28 | | | 35 | 37 | 28 | 29·80 | 30·00 | | | 9 | 9 | NNW | W | Fine | | |
| 29 | | | 30 | 32 | 31 | 30·14 | 30·07 | | | 9 | 12 | W | SSW | Fine | | |
| 30 | ◑ | | 33 | 39 | 28 | 30·00 | 29·90 | | | 9 | 9 | W | W | Fine | Rain | Clo. |
| 31 | | | 29 | 31 | 25 | 30·04 | 30·01 | | | 15 | 20 | WNW | N | Fog | | |
| Jan. | | | | | | | | | | | | | | | | |
| 1 | | | 27 | 30 | 30 | 30·02 | 30·01 | | | 15 | 15 | N | N | Fog | Fine | |
| 2 | | | 32 | 34 | 27 | 30·01 | 29·81 | | | 17 | 15 | NE | E | Snow | Clo. | |
| 3 | | | 30 | 31 | 34 | 29·70 | 29·56 | | | 17 | 22 | E | SE | Clo. | Rain | |
| 4 | | | 37 | 40 | 34 | 29·50 | 29·51 | | | 17 | 20 | S | S | Fine | | |
| 5 | | | 37 | 49 | 34 | 29·50 | 29·60 | | | 19 | 20 | SE | S | Rain | | |
| 6 | ● | | 37 | 39 | 35 | 29·96 | 30·16 | | | 15 | 17 | SE | SW | Fine | | Clo. |
| 7 | | | 38 | 43 | 32 | 29·10 | 29·87 | | | 17 | 22 | SW | SSW | Fine | | Rain |
| 8 | | | 39 | 42 | 35 | 30·16 | 30·21 | | | 12 | 12 | SW | W | Fine | Clo. | Fine |
| 9 | | | 39 | 45 | 43 | 29·96 | 29·88 | | | 18 | 15 | SW | SW | Rain | | |
| 10 | | | 46 | 51 | 48 | 29·80 | 29·71 | | | 21 | 22 | SW | SW | Fine | Rain | Clo. |
| 11 | | | 49 | 49 | 44 | 29·69 | 29·58 | | | 15 | 19 | SSW | SW | Fine | Rain | Clo. |
| 12 | | | 45 | 49 | 43 | 30·03 | 29·98 | | | 19 | 17 | W | WSW | Fine | | Rain |
| 13 | | | 47 | 52 | 47 | 29·97 | 29·9[illegible] | | | 15 | 15 | W | W | Fine | Clo. | Rain |
| 14 | ○ | | 49 | 49 | 44 | 29·80 | 29·70 | | | 19 | 16 | W va. | W va. | Rain | | Clo. |
| 15 | | | 48 | 52 | 49 | 29·69 | 29·75 | | | 15 | 20 | W va. | W va. | Rain | | |
| 16 | | | 49 | 54 | 42 | 29·61 | 29·55 | | | 19 | 19 | W va. | W va. | Rain | | |
| 17 | | | 42 | 42 | 39 | 29·78 | 29·94 | | | 12 | 10 | W va. | W va. | Fine | | |
| 18 | | | 41 | 44 | 30 | 29·95 | 30·21 | | | 10 | 9 | W | WSW | Fine | | |
| 19 | | | 34 | 39 | 33 | 30·55 | 30·51 | | | 7 | 8 | WSW | W | Fine | | |

The thermometer having been often below the freezing-point, the quantity of rain for the month of December cannot be given.

### REPORT OF DISEASES.

NOTWITHSTANDING the mildness of the season, pulmonary complaints have at last become frequent. Catarrhal fever has been almost epidemic, and very tedious, but not severe. Phthisical patients, in the advanced stages of the disease, but who, under a severer winter, might have vegetated in their chambers till spring, are likely to be cut off earlier, by being enabled to breathe the external air, and to engage in exercise, however light. We know not whether the same has occurred to others, but to the reporter these subjects have not seemed to show that complacency, or to indulge those flattering hopes, which are in general so characteristic of this interesting complaint.

A case of Tic Douloureux, at which we slightly hinted in our notice of Mr. Baillie's performance, has given us so good an opinion of his remedy, that we cannot fail to report the yet imperfect history. The subject has been four years afflicted, during which time she has applied to various persons,—private practitioners and medical officers of public charities. As far as could be collected, every remedy had been tried, excepting the operation. She began with only two grains of the extract of belladonna, procured from Apothecaries' Hall. This produced so violent an effect, as to alarm the lady in whose service the patient was: it was, therefore, intermitted for a time. The patient had, however, found a relief of which she had despaired; she was now advised to take only one grain whenever she apprehended the slightest return, and to continue the dose every hour as long as she found such relief necessary, and could venture to use it. The success has been such as to promise a complete cure: she has never had occasion for more than a second or third grain.

A tedious case of Gonorrhœa benigna has at length yielded to an injection of red port-wine and water. There is something so whimsical in these complaints, that we cannot have on our list too great a variety of remedies, especially as our young patients are frequently very importunate under this inconvenience, however trifling.

